# MDM2 promotes cancer cell survival through regulating the expression of HIF-1α and pVHL in retinoblastoma

**DOI:** 10.3389/pore.2023.1610801

**Published:** 2023-01-19

**Authors:** Shouhua Zhang, Hongyan Xu, Weiming Li, Jianfeng Ji, Qifang Jin, Leifeng Chen, Qiang Gan, Qiang Tao, Yong Chai

**Affiliations:** ^1^ Department of General Surgery, Jiangxi Provincial Children’s Hospital, Nanchang, Jiangxi, China; ^2^ Department of Ultrasound, Joint Support Forces of the Chinese People’s Liberation Army 908 Hospital, Nanchang, Jiangxi, China; ^3^ Department of General Surgery, Second Affiliated Hospital of Nanchang University, Nanchang, Jiangxi, China; ^4^ Department of Ophthalmology, Jiangxi Provincial Children’s Hospital, Nanchang, Jiangxi, China

**Keywords:** HIF-1α, retinoblastoma, MDM2, pVHL, hypoxia

## Abstract

Hypoxia is an important tumor feature and hypoxia-inducible factor 1 (HIF-1) is a master regulator of cell response to hypoxia. Mouse double minute 2 homolog (MDM2) promotes cancer cell survival in retinoblastoma (RB), with the underlying mechanism remaining elusive. In this study, we investigated the role of MDM2 and its relation to HIF-1α in RB. Expression analysis on primary human RB samples showed that MDM2 expression was positively correlated with that of HIF-1α while negatively correlated with von Hippel-Lindau protein (pVHL), the regulator of HIF-1α. In agreement, RB cells with MDM2 overexpression showed increased expression of HIF-1α and decreased expression of pVHL, while cells with MDM2 siRNA knockdown or MDM2-specific inhibitor showed the opposite effect under hypoxia. Further immuno-precipitation analysis revealed that MDM2 could directly interact with pVHL and promotes its ubiquitination and degradation, which consequently led to the increase of HIF-1α. Inhibition of MDM2 and/or HIF-1α with specific inhibitors induced RB cell death and decreased the stem cell properties of primary RB cells. Taken together, our study has shown that MDM2 promotes RB survival through regulating the expression of pVHL and HIF-1α, and targeting MDM2 and/or HIF-1α represents a potential effective approach for RB treatment.

## Introduction

Retinoblastoma (RB) is one of the most common childhood intraocular malignancies, with approximately 8,000 new cases each year and a survival rate of less than 30% (1). The majority of the RB cases are caused by the biallelic loss of retinoblastoma gene (RB1) (2). Inactivation of RB1 disrupts genomic stability in various forms, resulting in the dysregulation of gene expression and pathway activation (3). The genome instability and dysregulated gene expression and pathway activation accumulate and together promote tumorigenesis (4). So far, a long list of dysregulated genes has been identified in RB, however, their roles in RB development remain largely unknown (5).

Mouse double minute 2 homolog (MDM2) is an oncogene, which is overexpressed in various cancers and negatively regulates the tumor suppressor p53 (6). However, MDM2 has also been shown to promote cell survival in a p53-independent manner (7, 8). In RB, MDM2-induced cancer survival is p53-independent but the exact mechanism remains elusive (8, 9). Previous study has shown that MDM2 can promote hypoxia-inducible factor-1 (HIF-1) activation through a p53-independent pathway in colon carcinoma cells during hypoxia (7). However, whether MDM2 functions *via* HIF-1 in RB remains to be explored.

Hypoxia is an important tumor feature caused by an inadequate supply of oxygen due to uncontrolled tumor cell growth (10). HIF-1, a master regulator of cell response to hypoxia, contains 2 subunits, HIF-1α and HIF-1β (7, 11). While HIF-1β is constitutively expressed, HIF-1α is regulated by von Hippel-Lindau protein (pVHL), a E3 ubiquitin ligase (12). During hypoxia, HIF-1α accumulates and forms heterodimers with HIF-1β, which subsequently leads to the activation of cell survival genes (7, 11). A clinicopathological study has demonstrated that expression of HIF‐1α was observed in 83% (35/42) of RB tumors, which clearly shows that hypoxic pathway is activated in majority of the RB tumors (13). A connection between MDM2 and HIF-1 is still remained to established.

In this study, we aimed to investigate the expression and activity of MDM2 in RB cell survival and its relation to HIF-1 pathway. Our data showed that MDM2 promoted RB cell survival and such activity of MDM2 was through its ability to increase HIF-1α expression by degrading pVHL under hypoxic conditions.

## Materials and methods

### Ethical statement

All protocols involving human subjects and samples were approved by the institute ethical review board (Approval No. JXSETYY-YXKY-20180010) and performed according to the Declaration of Helsinki. In addition, informed consent has been obtained from the participants involved. The clinicopathological data for the participated patients were listed in Supplementary Table S1.

### Cells, plasmids and siRNAs

The human retinoblastoma cell lines Y79 and WERI were purchased from cell bank of Chinese Academy of Sciences, and were cultured in RPMI1640 with 10% FBS. Plasmids encoding human MDM2 (Cat. No. HG11206-UT) and Flag-tagged human MDM2 (Cat. No. HG11206-NF) were both purchased from SinoBiological. siRNA targeting MDM2 (Cat. No. AM51331) and negative control siRNA (Cat. No. 4390844) were both purchased from ThermoFisher Scientific.

### Dissection and isolation of human primary retinoblastoma cells

Primary RB cells were isolated as previously described with modifications (14). In brief, fresh human RB tumor samples were washed three times in ice-cold phosphate buffer saline (PBS). Dissociation into single cells was achieved by incubation in 0.25% trypsin-0.01% EDTA (Invitrogen, ThermoFisher Scientific) at 37°C for 10 min and gentle pipetting, then trypsin was neutralized with addition of fetal bovine serum (FBS, Gibco ThermoFisher Scientific). Dissociated cells were filtered with 70 μm cell strainer (Corning) and centrifuged at 1,000 rpm for 5 min, and followed by resuspension in serum free Dulbecco’s modified Eagle’s medium (DMEM)/F12 medium (Gibco, ThermoFisher Scientific) supplemented with 20 ng/mL recombinant human leukemia inhibitory factor (Chemicon). Isolated cells were maintained at 37°C, 5% CO_2_ under a humidified atmosphere.

### Cell treatments

For hypoxia treatment, cells were cultured in a hypoxia chamber (Galaxy® 48 R CO2, Eppendorf) with conditions of 0.2% oxygen and 5% carbon dioxide at 37°C. For inhibitor treatment, cells were treated with 1 µM MDM2 inhibitor SP-141 (Cat. No. 5332; Tocris Bioscience), or 5 µM HIF-1α inhibitor 2-MeOE2 (Cat. No. S1233; Selleck Chemicals) or both for 48 h at 37°C.

### Transfection

To overexpress MDM2, cells were transfected with plasmid encoding either untagged or Flag-tagged human MDM2 using Lipofectamine 2000 (Invitrogen, ThermoFisher Scientific), according to the manufacturer’s instructions. To knockdown MDM2 expression, cells were transfected with MDM2 siRNA using Lipofectamine™ RNAiMAX (Invitrogen, ThermoFisher Scientific), according to the manufacturer’s instructions.

### pVHL stability analysis

The impact of MDM2 overexpression on pVHL half life was assessed as previously described with modifications (15). In brief, cells were first transfected with MDM2 plasmid or empty vector (control), and then cells were treated with 50 μg/mL cycloheximide (CHX) for 1, 2, 4, 6 and 8 h under hypoxia. At these indicated time points, cells were harvested and the expression of pVHL was analyzed by western blot.

### Co-immunoprecipitation (Co-IP)

Co-IP was performed as previously described with modifications (16). In brief, cells were first transfected with Flag-MDM2 plasmid or empty vector (control) for 24 h and then cells were lysed with IP Lysis Buffer (Pierce, ThermoFisher Scientific) supplemented with Halt™ Protease Inhibitor Cocktail (ThermoFisher Scientific). Cleared lysed samples were then incubated with Dynabeads Protein G (Cat. No. 10003D; Invitrogen, ThermoFisher Scientific) for 1 h at room temperature to eliminate non-specific binding. Subsequently, samples were incubated with Dynabeads coating with anti-pVHL antibody (Cat. No. 24756-1-AP; ProteinTech), or control IgG (Cat. No. 30000-0-AP; ProteinTech) for 1 h at room temperature with end-to-end rotation. Following incubation, the Dynabead-Ab-Ag complex was then washed with PBST for 3 times and the immunoprecipitated protein was then eluted with SDS-PAGE loading buffer and heated at 90°C for 10 min.

### Western blotting

Western blotting analysis was performed as previously described with modifications (17). In brief, cells were first lysed with IP Lysis buffer (Pierce, ThermoFisher Scientific) supplemented with Halt™ Protease Inhibitor Cocktail (ThermoFisher Scientific) and then protein concentration was determined by BCA Protein Assay (ThermoFisher Scientific) according to the manufacturer’s instructions. Following quantification, 20 µg total protein was mixed with SDS-PAGE loading buffer, heated at 90°C for 10 min and separated by SDS-PAGE gel. For Co-IP samples, they were directly loaded onto SDS-PAGE gel for separation. Resolved protein was subsequently transferred onto an Immobilon-P PVDF membrane (Millipore, Merck). After blocking with 5% nonfat milk in PBST at room temperature for 1 h, the membrane was then sequentially incubated with primary antibodies and HRP-conjugated secondary antibodies. Primary antibody incubation was performed at 4°C overnight, while secondary incubation was performed at room temperature for 1 h. Following incubations, the membrane was extensively washed with PBST and immunobands were detected by LumiGLO reagent (Cell Signaling Technology). The following primary antibodies were used: rabbit anti-human MDM2 antibody (Cat. No. 27883-1-AP; ProteinTech), rabbit anti-human HIF-1α antibody (Cat. No. 20960-1-AP; ProteinTech), rabbit anti-pVHL antibody (Cat. No. 24756-1-AP; ProteinTech), rabbit anti-human β-actin antibody (Cat. No. 81115-1-RR; ProteinTech), mouse anti-Ubiquitin (P4D1) (Cat. No. 3936; Cell Signaling Technology), mouse anti-Flag (DYKDDDDK) antibody (Cat. No. 66008-4-Ig; ProteinTech). The following secondary antibodies were used: mouse anti-rabbit IgG-HRP (Cat. No. sc-2357; Santa Cruz) and HRP-conjugated Affinipure Goat Anti-Mouse IgG(H + L) (Cat. No. SA00001-1; ProteinTech).

### Real-time PCR

Total RNA was first extracted from cultured cells using the RNeasy Mini Kit (Qiagen) and then transcribed into cDNA using the iScript Select cDNA synthesis kit (Bio-Rad). The transcribed cDNA was subsequently used as template to amplify target gene sequences, using the iQ SYBR Green Supermix (Bio-Rad) on the iCycler iQ Real-Time Detection System (Bio-Rad). The β-actin mRNA was used as an internal control and the relative expression of each target gene was calculated using the 2^−ΔΔCt^ formula. The following primer pairs were used in the current study: hif-1α: forward primer: 5′-TAT​GAG​CCA​GAA​GAA​CTT​TTA​GGC-3′ and reverse primer: 5′-CAC​CTC​TTT​TGG​CAA​GCA​TCC​TG-3′, mdm2: forward primer: 5′-TGT​TTG​GCG​TGC​CAA​GCT​TCT​C-3′ and reverse primer: 5′-CAC​AGA​TGT​ACC​TGA​GTC​CGA​TG-3′, VHL: forward primer: 5′-GAC​ACA​CGA​TGG​GCT​TCT​GGT​T-3′ and reverse primer 5′-ACA​ACC​TGG​AGG​CAT​CGC​TCT​T-3′, and β-actin: forward primer: 5′-AAG​CAG​GAG​TAT​GAC​GAG​TCC​G-3′, and reverse primer 5′-GCC​TTC​ATA​CAT​CTC​AAG​TTG​G-3’. All primers were purchased from OriGene.

### Cell viability assay

Cell viability was assessed using a commercial CellTiter-Glo Luminescent Cell Viability Assay Kit (Promega), according to the manufacturer’s instructions. Briefly, cells pre-seeded at 1 × 10^4^ cells/well in 96-well plates were first treated with 1 µM MDM2 inhibitor SP-141 (Cat. No. 5332; Tocris Bioscience), or 5 µM HIF-1α inhibitor 2-MeOE2 (Cat. No. S1233; Selleck Chemicals) or both for 48 h at 37°C under normoxic or hypoxic conditions. After treatment, equal volume of CellTiter-Glo reagent was added to each well and the plate was incubated for 2 min on an orbital shaker to induce cell lysis. Then the plate was kept at room temperature for 10 min to stabilize luminescent signal and luminescence was subsequently recorded. Cell viability was calculated with cells with mock treatment being considered as 100% viable.

### Apoptosis assay

Cell apoptosis was detected using a commercial Caspase-Glo 3/7 Assay System (Promega), according to the manufacturer’s instructions. In brief, cells in 96-well plates were first treated as detailed in the Cell viability assay above, and then equal volume of Caspase-Glo 3/7 reagent was added to each well. The plate was then mixed by gentle shaking on an orbital shaker for 30 s, followed by incubation at room temperature for 30 min. Luminescence was finally recorded by a microplate reader.

### RB cancer stem cell (CSC) culture

RB CSCs were generated and cultured as previously described with modifications (14). In brief, primary RB cells from 5 patients were pooled and cultured in serum-free CSC culture medium (DMEM/F12 medium supplemented with 20 ng/mL recombinant human EGF (R&D systems), 20 ng/mL recombinant human FGF2 (StemCell), 1x B27 Plus supplement (Gibco, ThermoFisher Scientific), 1x Penicillin-Streptomycin-Glutamine (Gibco, ThermoFisher Scientific), 5 μg/mL heparin (StemCell), and 20 ng/mL recombinant human LIF (SinoBiological). Cells were subcultured every 3 days until single cells died out and survived cells formed CSC-like solid spheroids, a key characteristic of CSCs. The volume of the spheroids was measured as previously described using the formula: $Volume={\left(mean\;diameter\right)}^{3}\times \frac{\pi }{6}$ (18). The mean diameter of the spheroids was defined as the mean value of the longest and shortest diameters in this study.

### Statistical analysis

Data were expressed as mean +/− standard deviation (SD) in the current study and statistical analyses were performed with GraphPad Prism (version 7.0.4, GraphPad). Simple linear regression was used to assess the correlation between two variables. Mann-Whitney test was used to compare the difference between two groups, while Kruskall-Wallis test plus Dunn’s multiple comparisons test were used to compare the difference among three or more groups. A *p*-value less than 0.05 was considered statistically significant.

## Results

### The expression of MDM2, HIF-1α and pVHL in human primary RB cells

**FIGURE 1:**
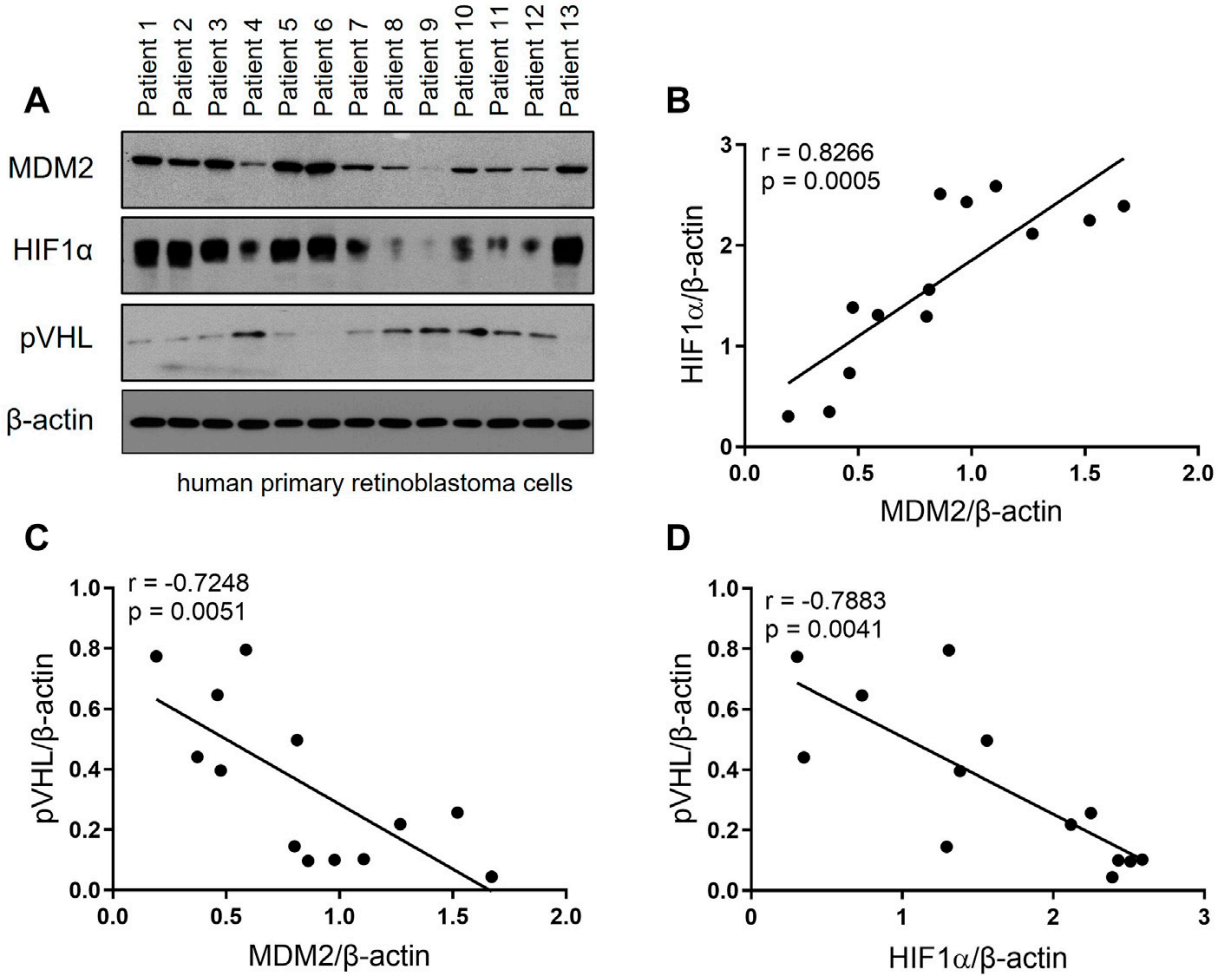
The expression of MDM2, HIF-1α and pVHL in human primary RB. **(A)** Primary RB cells were isolated from 13 patients and the levels of MDM2, HIF-1α, and pVHL were evaluated by western blot. **(B–D)** The band intensity of the western blot was quantitated by ImageJ, and then the correlation between **(B)** MDM2 and HIF-1α, **(C)** MDM2 and pVHL and **(D)** HIF-1α and pVHL was analyzed by simple linear regression. The goodness of fit, r and *p* values, were shown in each correlation analysis.

To investigate the expression profile of MDM2, HIF-1α and pVHL in RB patients, primary RB cells from 13 RB patients were harvested and the expression of these 3 proteins were determined by Western blot. As shown in Figure 1A, all 3 proteins were successfully detected in the primary RB cells. Correlation analyses were performed to further explore the relation of the 3 proteins. Our data showed that MDM2 expression was positively correlated with that of HIF-1α, while the expression of MDM2 and pVHL, as well as that of HIF-1α and pVHL were negatively correlated (Figures 1B–D). These results indicate a correlation among MDM2, HIF-1α and pVHL.

### MDM2 regulates the expression of HIF-1α and pVHL under hypoxia

**FIGURE 2:**
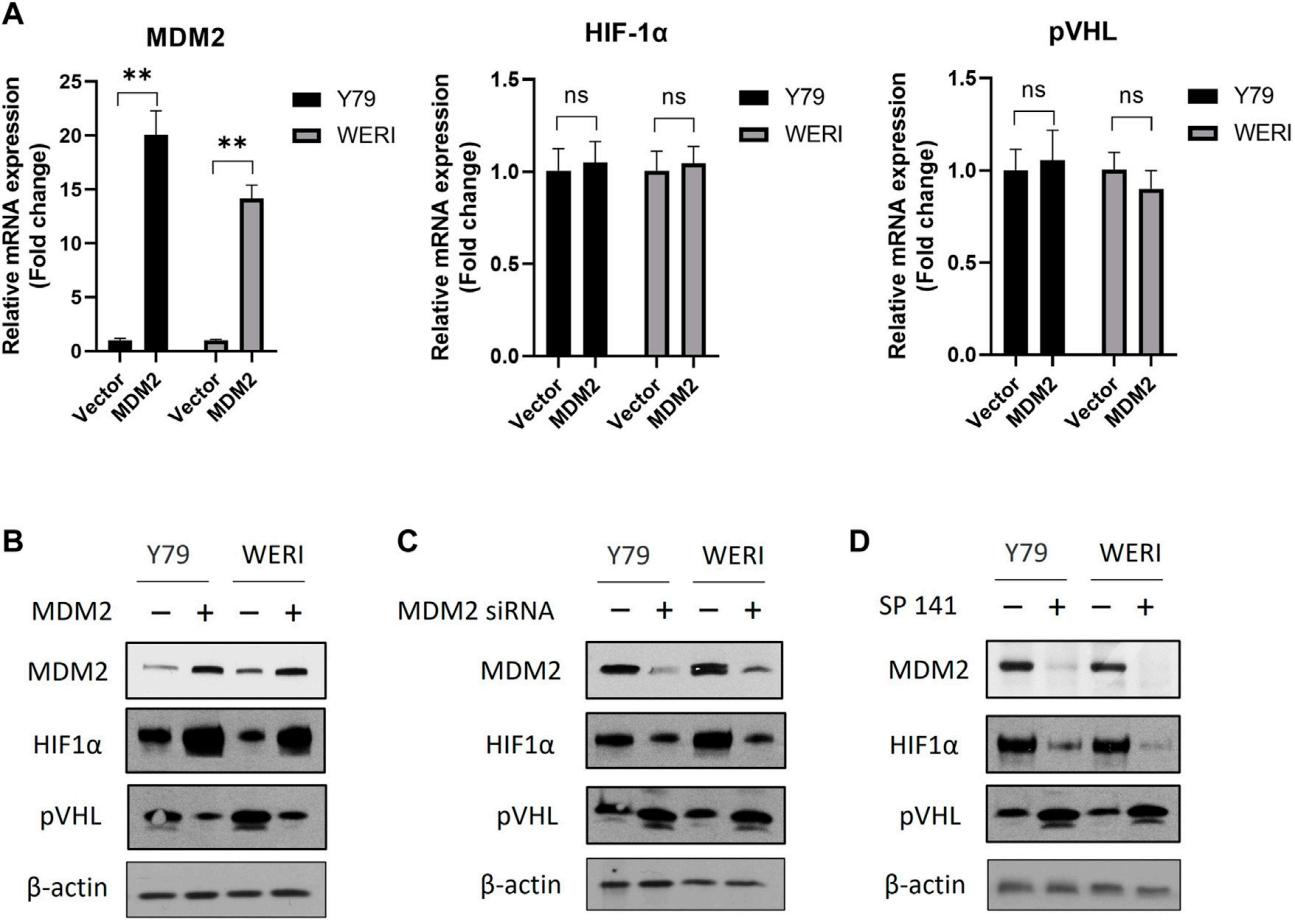
MDM2 regulates the expression of HIF-1α and pVHL under hypoxia. **(A, B)** MDM2 was first overexpressed in Y79 and WERI cells, and **(A)** the mRNA and **(B)** protein levels of MDM2, HIF-1α, and pVHL in hypoxia were evaluated by RT-PCR and western blot, respectively. **(A)** Data shown are mean +/− SD of 3 independent experiments. n.s, not statistically significant; ***p < 0.001. **(B)** One representative result is shown. **(C)** MDM2 was first knocked down by siRNA in Y79 and WERI cells, and then the protein levels of MDM2, HIF-1α, and pVHL in hypoxia were evaluated by western blot. One representative result is shown. **(D)** Y79 and WERI cells were first treated with MDM2 inhibitor SP 141 under hypoxia, and then the protein levels of MDM2, HIF-1α, and pVHL were evaluated by western blot. One representative result out of 3 is shown.

To investigate the impact of MDM2 on the expression of HIF-1α and pVHL, RB cell lines Y79 and WERI were first overexpressed with MDM2 under hypoxia, and then the mRNA and protein levels of HIF-1α and pVHL were determined by real time PCR and western blot, respectively. Our results showed that MDM2 overexpression did not show apparent impact on the mRNA levels of HIF-1α and pVHL in either Y79 or WERI cells (Figure 2A). However, a considerable increase of HIF-1α while a decrease of pVHL on protein level was observed in the cells overexpressed with MDM2 (Figure 2B; Supplementary Figure S1A). These data indicate that MDM2 could regulate the expression of HIF-1α and pVHL on the protein level, but not on the mRNA level.

To further confirm the regulatory role of MDM2 on HIF-1α and pVHL, Y79 and WERI cells under hypoxia were treated with MDM2 siRNA or a MDM2 specific inhibitor SP141. As shown in Figure 2C and Supplementary Figure S1B, knockdown of MDM2 with siRNA decreased the expression of HIF-1α while increased the expression of pVHL in both Y79 and WERI cells. A similar result was also observed in cells treated with SP141 (Figure 2D; Supplementary Figure S1C). Namely, the MDM2-specific inhibitor reduced MDM2 and HIF-1α expression while increased pVHL expression (Figure 2D; Supplementary Figure S1C). Taken together, our data here indicate that MDM2 may have a regulatory effect on the expression of HIF-1α and pVHL, either through direct or indirect mechanisms.

### MDM2 directly interacts with pVHL and promotes the ubiquitination of pVHL

**FIGURE 3:**
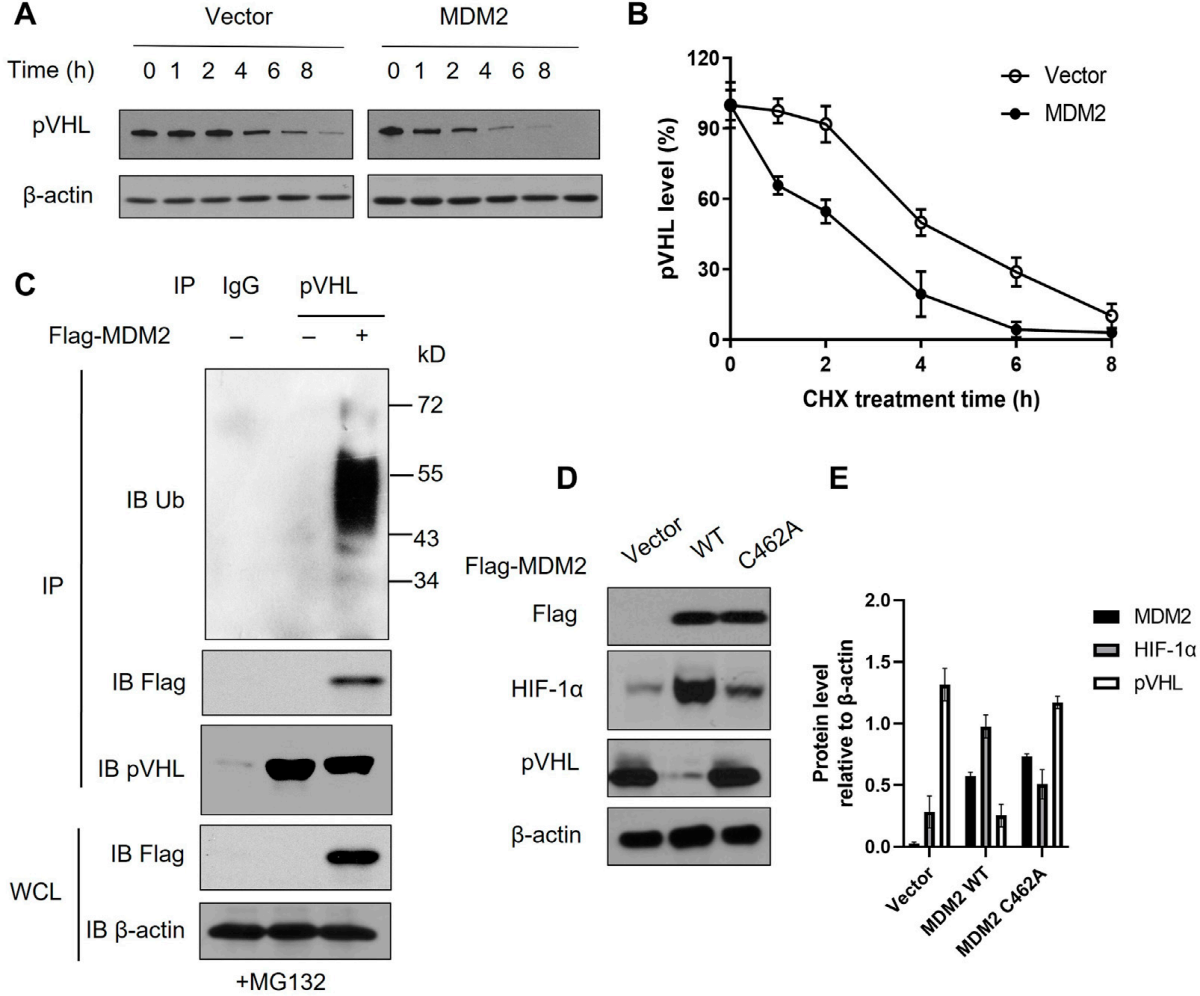
MDM2 directly interacts with pVHL and promotes the ubiquitination of pVHL. **(A)** Y79 cells with or without MDM2 overexpression was treated with cycloheximide (CHX) for various time periods and then the level of pVHL in cells was evaluated by western blot. One representative result out of 3 is shown. **(B)** The western blot results were quantitated by ImageJ and plotted as mean +/− SD of 3 independent experiments. **(C)** Y79 cells were first overexpressed with MDM2, and then cells were lysed and pVHL was immune-precipitated by pVHL antibody. MDM2, pVHL and ubiquitinated pVHL in the complex were examined by western blot. One representative result out of 3 replicates is shown. IP: immunoprecipitation; WCL: whole cell lysate; IB: immunoblotting. **(D, E)** Y79 cells were first overexpressed with wild type (WT) or mutated (C462A) MDM2 under hypoxia and then cells were lysed and the expression of MDM2, HIF-1α, and pVHL was assessed by Western blot. **(D)** One representative result out of 3 replicates is shown. **(E)** The western blot results were quantitated by ImageJ and plotted as mean +/− SD of 3 independent experiments.

Our data above showed that MDM2 expression was correlated with both HIF-1α and pVHL. Given that HIF-1α is regulated by pVHL, it is likely that MDM2 affected the expression of HIF-1α *via* its regulation on pVHL expression (12). Interaction between MDM2 and pVHL has been previously described and we suspected if MDM2, with its E3 ubiquitin ligase activity, could promote pVHL degradation through ubiquitination (19). To test our hypothesis, we first overexpressed MDM2 in Y79 cells and examined the half-life of pVHL under hypoxia. Our data showed that pVHL had an accelerated degradation pattern in cells with MDM2 overexpression comparing to cells without MDM2 overexpression (Figures 3A, B; Supplementary Figures S2A, B). To further explore if there is direct interaction between MDM2 and pVHL, endogenous pVHL was immunoprecipitated by anti-pVHL antibody in Y79 cells and the presence of MDM2 in the immunoprecipitated product was determined by western blot. As we suspected, overexpressed MDM2 was co-precipitated with pVHL, indicating a direct interaction between the 2 proteins (Figure 3C; Supplementary Figure S2C). Moreover, the blotting with an anti-Ub antibody further demonstrated that the ubiquitination of pVHL was significantly enhanced by MDM2 overexpression (Figure 3C; Supplementary Figure S2C). To further confirm the causal relationship between MDM2 overexpression-induced pVHL ubiquitination and the increase of HIF-1α expression, we generated a mutated version of MDM2, MDM2^C462A^. The introduction of this mutation altered a structural-crucial zinc coordinating cysteine in MDM2, and consequently abolished the E3 ubiquitin ligase activity of MDM2 and its ability to ubiquitinate its substrates like pVHL (20,21,22). Our results showed that the expression of HIF-1α was increased in cells transfected with wild type MDM2, but such increase was not detected in cells transfected with MDM2^C462A^, confirming that the ubiquitin ligase activity of MDM2 on pVHL was involved in the regulation of HIF-1α expression (Figures 3D, E). Taken together, these results indicate that MDM2 regulated pVHL through promoting ubiquitination of pVHL, which consequently led to the upregulation of HIF-1α.

### Inhibition of MDM2 and HIF-1α induces apoptosis in RB cells under hypoxic condition

**FIGURE 4:**
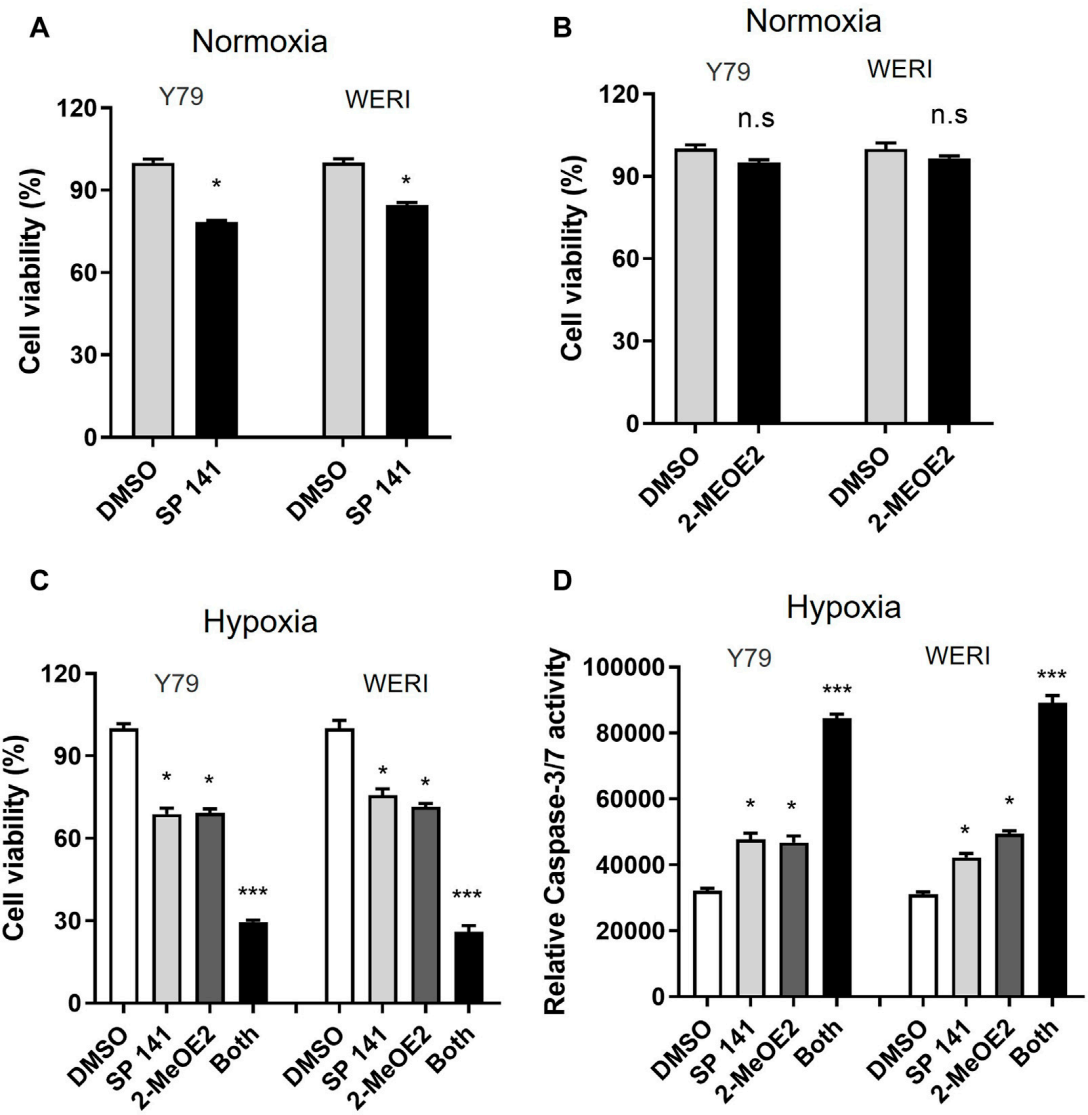
Inhibition of both MDM2 and HIF-1α induces apoptosis in RB cells under hypoxic conditions. **(A, B)** Y79 and WERI cells were treated by **(A)** MDM2 inhibitor SP 141 or **(B)** HIF-1α-specific inhibitor 2-MeOE2 under normoxia, and cell viability was measured by CellTiter-Glo assay. Data shown are mean +/− SD of 3 independent experiments. n.s, not statistically significant; *p < 0.05. **(C, D)** Y79 and WERI cells were first treated with MDM2 inhibitor SP 141 and/or HIF-1α inhibitor 2-MeOE2 under hypoxia, and then cell viability was measured by **(C)** CellTiter-Glo assay and **(D)** Caspase 3/7 activity, respectively. Data shown are mean +/- SD of 3 independent experiments. *p < 0.05; ***p < 0.001.

To investigate the effect of MDM2 and HIF-1α on RB cell viability under hypoxia, RB cells Y79 and WERI were treated with MDM2-specific inhibitor SP141 and/or HIF-1α-specific inhibitor 2-MeOE2 under normoxia or hypoxia. Under normoxia, the proliferation of Y79 and WERI cells upon SP141 treatment was dropped to 78.30% and 84.60%, respectively (Figure 4A). However, under hypoxia, both cell lines showed further decrease in cell proliferation after SP141 treatment, with Y79 being 68.9% and WERI being 75.75% of control, respectively (Figure 4C). Treatment of 2-MeOE2 did not show apparent impact on cell viability under normoxia (Figure 4B), but significantly reduced cell viability under hypoxia (Figure 4C). Moreover, a further reduction in cell viability was observed when cells were treated with both SP141 and 2-MeOE2 under hypoxia (Figure 4C). In accordance, cell apoptosis, as indicated by Caspase-3/7 activity, was increased when cells were treated with SP141 or 2-MeOE2, and similarly a further increase was observed when a combination of SP141 and 2-MeOE2 was used (Figure 4D). Taken together, our data here indicate that both MDM2 and HIF-1α are important for RB cell survival under hypoxia and inhibition of these 2 proteins could result in enhanced cell death.

### Inhibition of MDM2 and HIF-1α decreases the stem-cell properties of primary retinoblastoma cells

Increasing evidence has confirmed that a subset of cells, existed in many cancer types, exhibit capacity to rapidly proliferate and self-renewal, now known as cancer stem cells (CSCs) (23). CSCs are thought to play an important role in cancer initiation and progression, which is relevant to tumor persistence to treatment. Previous study has shown that MDM2 is required for the efficient generation of induced pluripotent stem cells (iPSCs) from murine embryonic fibroblasts, in the absence of p53 (24). HIF activity is also critical in maintenance of cancer stem cells as well as differentiation and function of inflammatory cells (25). Since inhibition of MDM2 and HIF-1α could induce apoptosis in RB cells under hypoxia, next we sought to analyze whether inhibition of MDM2 and HIF-1α could regulate the stem-cell properties of RB.

**FIGURE 5:**
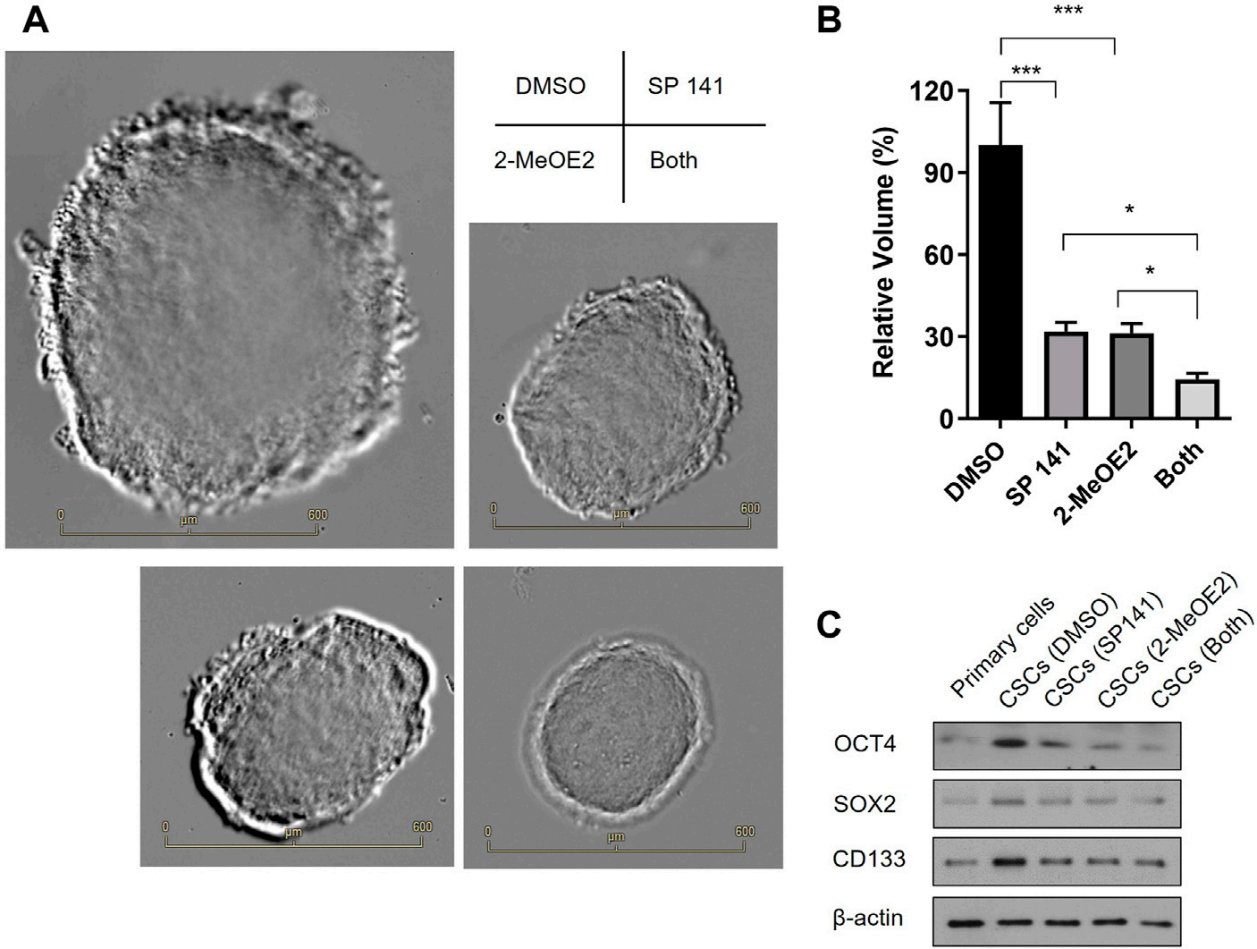
Inhibition of MDM2 and HIF-1α decreases the stem-cell properties of primary retinoblastoma cells. **(A)** RB CSCs were cultured in the presence or absence of MDM2 inhibitor SP 141 and/or HIF-1α-specific inhibitor 2-MeOE2 under hypoxia, and morphology of the CSC spheroids were captured under microscope. Representative images are shown. Bar = 600 µm. **(B)** The volumes of the CSC spheroids were calculated and differences among groups were compared (*n* = 5 spheroids per treatment). *p < 0.05; ***p < 0.001. **(C)** The expression of CSC markers OCT4, SOX2 and CD133 was determined by Western blot. One representative result out of 3 replicates is shown.

Primary cells were isolated from fresh human RB tissues after enucleation and cultured in serum-free conditions in the presence of DMSO, MDM2 inhibitor SP 141 and/or HIF-1α inhibitor 2-MeOE2 under hypoxia. As shown in Figure 5A, cells from all treatment groups formed stem-cell like spheroids, a characteristic morphology of CSCs. However, the spheroid sizes were distinctively different among treatments. In detail, the largest spheroids were observed in the DMSO control group, while the size of these spheroids were reduced by 70% in SP 141 and 2-MeOE2 treated groups (Figures 5A, B). Moreover, a further reduction in size for the spheroids were seen in cells treated with both SP 141 and 2-MeOE2, with the mean volume being only 15% of those from the DMSO group (Figures 5A, B). The stemness of the formed CSCs were also investigated by assessing the expression of CSC markers OCT4, SOX2 and CD133. Similarly, treatment with SP141 or 2-MeOE2 alone reduced the expression of all 3 markers in comparison to DMSO treatment, and the treatment with SP141 and 2-MeOE2 together further reduced the expression of these markers (Figure 5C). Collectively, these data showed MDM2 and HIF-1α are of great importance in the generation of CSCs in RB and inhibition of these 2 proteins could significantly decrease the stem-cell properties of primary retinoblastoma cells.

## Discussion

Hypoxia, a condition of oxygen deficiency, is a common characteristic for all solid tumors and is also important hallmark of tumor feature (26, 27). Hypoxia is involved in a wide range of aspects in tumor biology, including cell proliferation, angiogenesis, invasion, and metastasis (28). It is well documented that HIFs are the master regulators of hypoxia in cells or tissue and that HIF-1α is regulated by pVHL in response to oxygen level, with several possible mechanisms being proposed (12, 29, 30). Recent studies have indicated the involvement of PI3K/AKT pathway and PKA pathway in HIF-1α phosphorylation and stabilization, and MAPK/ERK pathway in the nuclear accumulation of HIF-1α (31,32,33,34). In addition, reactive oxygen species (ROS) is evidenced to increase HIF-1α stability in some studies but to induce HIF-1α degradation through the ubiquitin proteasome pathway in other studies (31,35,36,37). In the current study, we have demonstrated that HIF-1α could be regulated by MDM2 through its regulatory role on the half-life of pVHL. Although it was not addressed in our current study, it would be interesting for further studies to investigate if one or more of the previously identified pathways on HIF-1α is involved in MDM2-mediated HIF-1α regulation.

CSCs are a type of poorly differentiated and highly tumorigenic cells that can survive within regions with extreme hypoxia (23). Due to their rapid proliferation rate and self-renewal, these cells have enhanced tumor-initiating capability and are more resistant to various anti-cancer therapies (26, 38). In our current study, we have confirmed that RB cells could acquire CSC features under hypoxia, and also that HIF-1α played a role in the establishment of these features. Moreover, we have also shown that targeting MDM2 and/or HIF-1α represents an effective approach in limiting CSC growth. Although further studies are required, it is warranted to investigate the anti-cancer value of drugs targeting MDM2 and/or HIF-1α in RB or even other solid tumor types.

MDM2 is an oncogene that is highly expressed in various tumor cells and it promotes cell survival mainly through the attenuation of p53-mediated tumor surveillance. In some cases like RB, however, a p53-independent mechanism has also been evidenced. While the p53-dependent pathway is well characterized, the p53-independent one is not yet fully studied. Increasing evidence has shown that MDM2 are involved in various aspects of cellular processes, including cell proliferation, differentiation, apoptosis and genome stability (39). MDM2 achieves functions through a wide spectrum of mechanisms, including ubiquitination of alternative targets, stimulation of transcription factors and regulation of mRNA stability (39). A previous study has revealed that MDM2 could upregulate the transcription and translation of MYCN, an essential component to RB cell growth and tumor formation (8). Another study has indicated the involvement of HIF-1 in MDM2-induced p53-independent cancer cell growth under hypoxia (7). In our study, we have confirmed that the MDM2 could promote cancer survival through HIF-1. Our study has also shown mechanism of such activity. Namely, MDM2 could directly bind to and ubiquitinate pVHL and consequently cause the increase of HIF-1α.

## Conclusion

In this present study, we have shown that MDM2 expression is positively correlated to HIF-1α while negatively correlated with pVHL in primary RB tissue. Under hypoxia, MDM2 binds to pVHL and promotes the degradation of the latter through ubiquitination, which leads to increased HIF-1α expression. Both MDM2 and HIF-1α are important for RB cell survival under hypoxia and inhibition of either one or both lead to reduced RB cell survival and CSC formation. Our findings in the current study not only provide a deeper understanding of RB cell survival under hypoxia, but also offer potential treatment targets for RB. As a result of our current work, new strategies targeting MDM2 and HIF-1α could be developed that can be used to treat RB in the future.

## Data Availability

The original contributions presented in the study are included in the article/Supplementary Material, further inquiries can be directed to the corresponding authors.

## References

[1] Rojanaporn D Boontawon T Chareonsirisuthigul T Thanapanpanich O Attaseth T Saengwimol D Spectrum of germline RB1 mutations and clinical manifestations in retinoblastoma patients from Thailand. Mol Vis (2018) 24:778–88. 30636860 PMC6300611

[2] Dimaras H Corson TW Cobrinik D White A Zhao J Munier FL Retinoblastoma Nat Rev Dis primers (2015) 1(1):1–23. 10.1038/nrdp.2015.21 PMC5744255 27189421

[3] Burkhart DL Sage J. Cellular mechanisms of tumour suppression by the retinoblastoma gene. Nat Rev Cancer (2008) 8(9):671–82. 10.1038/nrc2399 18650841 PMC6996492

[4] Dimaras H Corson TW. Retinoblastoma, the visible CNS tumor: A review. J Neurosci Res (2019) 97(1):29–44. 10.1002/jnr.24213 29314142 PMC6034991

[5] Kaewkhaw R Rojanaporn D. Retinoblastoma: Etiology, modeling, and treatment. Cancers (2020) 12(8):2304. 10.3390/cancers12082304 32824373 PMC7465685

[6] Freedman D Wu L Levine A. Functions of the MDM2 oncoprotein. Cell Mol Life Sci CMLS (1999) 55(1):96–107. 10.1007/s000180050273 10065155 PMC11146946

[7] Nieminen A-L Qanungo S Schneider EA Jiang B-H Agani FH. Mdm2 and HIF-1alpha interaction in tumor cells during hypoxia. J Cell Physiol (2005) 204(2):364–9. 10.1002/jcp.20406 15880652

[8] Qi DL Cobrinik D. MDM2 but not MDM4 promotes retinoblastoma cell proliferation through p53-independent regulation of MYCN translation. Oncogene (2017) 36(13):1760–9. 10.1038/onc.2016.350 27748758 PMC5374018

[9] Xu XL Fang Y Lee TC Forrest D Gregory-Evans C Almeida D Retinoblastoma has properties of a cone precursor tumor and depends upon cone-specific MDM2 signaling. Cell (2009) 137(6):1018–31. 10.1016/j.cell.2009.03.051 19524506 PMC5659855

[10] Boutrid H Pina Y Cebulla CM Feuer WJ Lampidis TJ Jockovich M-E Increased hypoxia following vessel targeting in a murine model of retinoblastoma. Invest Ophthalmol Vis Sci (2009) 50(12):5537–43. 10.1167/iovs.09-3702 19578014

[11] Wang GL Semenza GL. Purification and characterization of hypoxia-inducible factor 1 (∗). J Biol Chem (1995) 270(3):1230–7. 10.1074/jbc.270.3.1230 7836384

[12] Safran M Kaelin WG. HIF hydroxylation and the mammalian oxygen-sensing pathway. J Clin Invest (2003) 111(6):779–83. 10.1172/JCI18181 12639980 PMC153778

[13] Sudhakar J Venkatesan N Lakshmanan S Khetan V Krishnakumar S Biswas J. Hypoxic tumor microenvironment in advanced retinoblastoma. Pediatr Blood Cancer (2013) 60(10):1598–601. 10.1002/pbc.24599 23804414

[14] Tang Z Ma H Mao Y Ai S Zhang P Nie C Identification of stemness in primary retinoblastoma cells by analysis of stem-cell phenotypes and tumorigenicity with culture and xenograft models. Exp Cel Res (2019) 379(1):110–8. 10.1016/j.yexcr.2019.03.034 30935947

[15] Park KS Kim JH Shin HW Chung KS Im DS Lim JH E2-EPF UCP regulates stability and functions of missense mutant pVHL via ubiquitin mediated proteolysis. BMC Cancer (2015) 15:800. 10.1186/s12885-015-1786-8 26503325 PMC4624580

[16] Hu K Fu M Wang J Luo S Barreto M Singh R HSV-2 infection of human genital epithelial cells upregulates TLR9 expression through the SP1/JNK signaling pathway. Front Immunol (2020) 11:356. 10.3389/fimmu.2020.00356 32194565 PMC7065266

[17] Hu K McKay PF Samnuan K Najer A Blakney AK Che J Presentation of antigen on extracellular vesicles using transmembrane domains from viral glycoproteins for enhanced immunogenicity. J Extracellular Vesicles (2022) 11(3):e12199. 10.1002/jev2.12199 35233930 PMC8888812

[18] Zhang S Li N Sheng Y Chen W Ma Q Yu X Hepatitis B virus induces sorafenib resistance in liver cancer via upregulation of cIAP2 expression. Infect Agents Cancer (2021) 16(1):20–11. 10.1186/s13027-021-00359-2 PMC7988944 33757557

[19] Wolf ER Mabry AR Damania B Mayo LD. Mdm2-mediated neddylation of pVHL blocks the induction of antiangiogenic factors. Oncogene (2020) 39(29):5228–39. 10.1038/s41388-020-1359-4 32555333 PMC7368819

[20] Nomura K Klejnot M Kowalczyk D Hock AK Sibbet GJ Vousden KH Structural analysis of MDM2 RING separates degradation from regulation of p53 transcription activity. Nat Struct Mol Biol (2017) 24(7):578–87. 10.1038/nsmb.3414 28553961 PMC6205632

[21] Geyer RK Yu ZK Maki CG. The MDM2 RING-finger domain is required to promote p53 nuclear export. Nat Cel Biol (2000) 2(9):569–73. 10.1038/35023507 10980696

[22] Itahana K Mao H Jin A Itahana Y Clegg HV Lindström MS Targeted inactivation of Mdm2 RING finger E3 ubiquitin ligase activity in the mouse reveals mechanistic insights into p53 regulation. Cancer cell (2007) 12(4):355–66. 10.1016/j.ccr.2007.09.007 17936560

[23] Batlle E Clevers H. Cancer stem cells revisited. Nat Med (2017) 23(10):1124–34. 10.1038/nm.4409 28985214

[24] Wienken M Dickmanns A Nemajerova A Kramer D Najafova Z Weiss M MDM2 associates with polycomb repressor complex 2 and enhances stemness-promoting chromatin modifications independent of p53. Mol Cel (2016) 61(1):68–83. 10.1016/j.molcel.2015.12.008 PMC6284523 26748827

[25] Peng G Liu Y. Hypoxia-inducible factors in cancer stem cells and inflammation. Trends Pharmacological Sciences (2015) 36(6):374–83. 10.1016/j.tips.2015.03.003 PMC4461458 25857287

[26] Najafi M Farhood B Mortezaee K Kharazinejad E Majidpoor J Ahadi R. Hypoxia in solid tumors: A key promoter of cancer stem cell (CSC) resistance. J Cancer Res Clin Oncol (2020) 146(1):19–31. 10.1007/s00432-019-03080-1 31734836 PMC11804417

[27] Kim H Lin Q Glazer PM Yun Z. The hypoxic tumor microenvironment *in vivo* selects the cancer stem cell fate of breast cancer cells. Breast Cancer Res (2018) 20(1):16–5. 10.1186/s13058-018-0944-8 29510720 PMC5840770

[28] Hayashi Y Yokota A Harada H Huang G. Hypoxia/pseudohypoxia‐mediated activation of hypoxia‐inducible factor‐1α in cancer. Cancer Sci (2019) 110(5):1510–7. 10.1111/cas.13990 30844107 PMC6501028

[29] Ryan HE Poloni M McNulty W Elson D Gassmann M Arbeit JM Hypoxia-inducible factor-1alpha is a positive factor in solid tumor growth. Cancer Res (2000) 60(15):4010–5. 10945599

[30] Jaakkola P Mole DR Tian Y-M Wilson MI Gielbert J Gaskell SJ Targeting of HIF-α to the von Hippel-Lindau ubiquitylation complex by O2-regulated prolyl hydroxylation. Science (2001) 292(5516):468–72. 10.1126/science.1059796 11292861

[31] Corrado C Fontana S. Hypoxia and HIF signaling: One Axis with divergent effects. Int J Mol Sci (2020) 21(16):5611. 10.3390/ijms21165611 32764403 PMC7460602

[32] Kietzmann T Mennerich D Dimova EY. Hypoxia-inducible factors (HIFs) and phosphorylation: Impact on stability, localization, and transactivity. Front Cel Dev Biol (2016) 4:11. 10.3389/fcell.2016.00011 PMC4763087 26942179

[33] Xiao Y Peng H Hong C Chen Z Deng X Wang A PDGF promotes the warburg effect in pulmonary arterial smooth muscle cells via activation of the PI3K/AKT/mTOR/HIF-1α signaling pathway. Cell Physiol Biochem (2017) 42(4):1603–13. 10.1159/000479401 28738389

[34] Zhang Z Yao L Yang J Wang Z Du G. PI3K/Akt and HIF‑1 signaling pathway in hypoxia‑ischemia (Review). Mol Med Rep (2018) 18(4):3547–54. 10.3892/mmr.2018.9375 30106145 PMC6131612

[35] Shvetsova AN Mennerich D Kerätär JM Hiltunen JK Kietzmann T. Non-electron transfer chain mitochondrial defects differently regulate HIF-1α degradation and transcription. Redox Biol (2017) 12:1052–61. 10.1016/j.redox.2017.05.003 28531964 PMC5440747

[36] Semenza GL. Hypoxia‐inducible factors: Coupling glucose metabolism and redox regulation with induction of the breast cancer stem cell phenotype. EMBO J (2017) 36(3):252–9. 10.15252/embj.201695204 28007895 PMC5286373

[37] Lacher SE Levings DC Freeman S Slattery M. Identification of a functional antioxidant response element at the HIF1A locus. Redox Biol (2018) 19:401–11. 10.1016/j.redox.2018.08.014 30241031 PMC6146589

[38] Jeong H Kim S Hong B-J Lee C-J Kim Y-E Bok S Tumor-associated macrophages enhance tumor hypoxia and aerobic glycolysis. Cancer Res (2019) 79(4):795–806. 10.1158/0008-5472.CAN-18-2545 30610087

[39] Bohlman S Manfredi JJ. p53-independent effects of Mdm2. Mutant p53 and MDM2 in Cancer. Subcell Biochem (2014) 235–46. 10.1007/978-94-017-9211-0_13 25201198 PMC5507576

