# Supplementary material for: MDM2 promotes cancer cell survival through regulating the expression of HIF-1α and pVHL in retinoblastoma
Source: Pathol Oncol Res. 2023 Jan 19;29:1610801. doi: 10.3389/pore.2023.1610801 (PMC9892057; doi:10.3389/pore.2023.1610801)
Supplement: Supplementary file 1 [file DataSheet1.docx]

**Table S1. Patient clinicopathological characteristics**

| Parameter | | Value |
| --- | --- | --- |
| Total patients |  | 13 (100 %) |
| Gender | Male | 7 (53.8%) |
|  | Female | 6 (46.2%) |
| Age (month) | Median | 22 |
|  | Mean | 24 |
|  | Range | 7-64 |
| Laterality | Left | 8 (61.5%) |
|  | Right | 5 (38.5%) |
| RB stage * | E | 13 (100%) |

* RB stage was assessed according to

the International Classification of Retinoblastoma (ICRB).


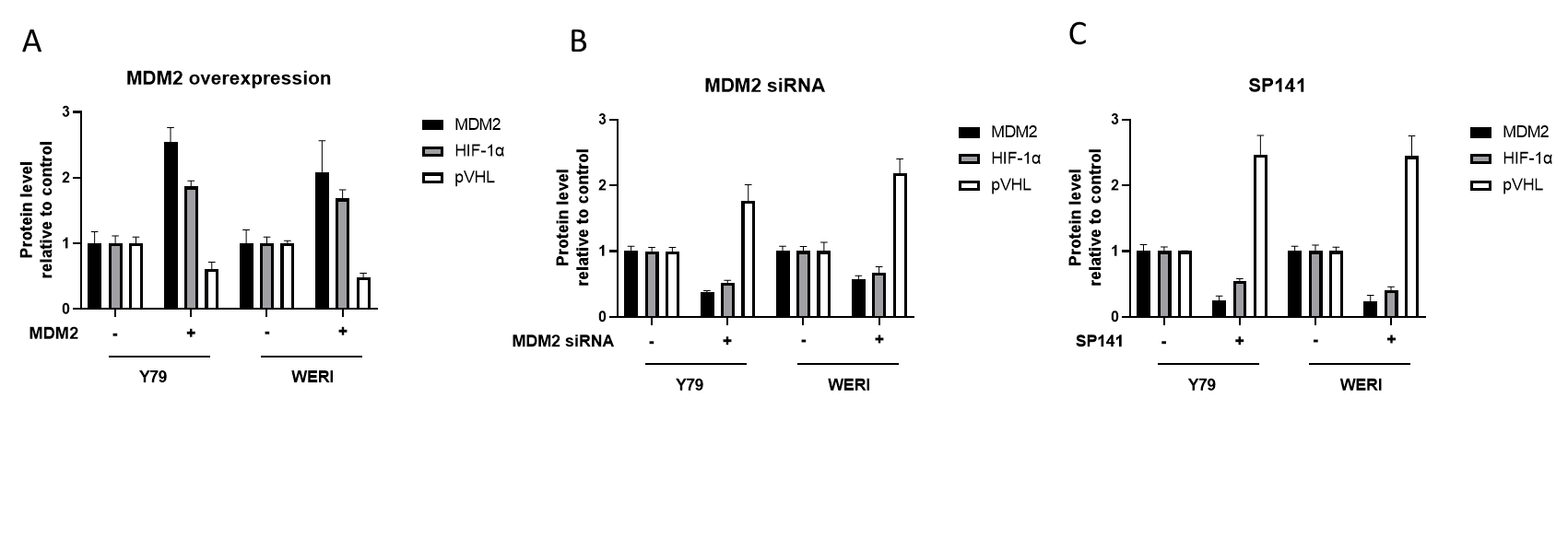


**Figure S1.** MDM2 regulates the expression of HIF-1α and pVHL under hypoxia. Y79 and WERI cells were either (A) transfected with MDM2, (B) MDM2 siRNA or (C) treated with MDM2 inhibitor SP141, and protein levels of MDM2, HIF-1α, and pVHL in hypoxia were evaluated by western blot. The western blot bands were quantified by Image J and the expression of each protein was normalized to that of the control group. Data shown are mean +/- SD of 3 independent experiments.


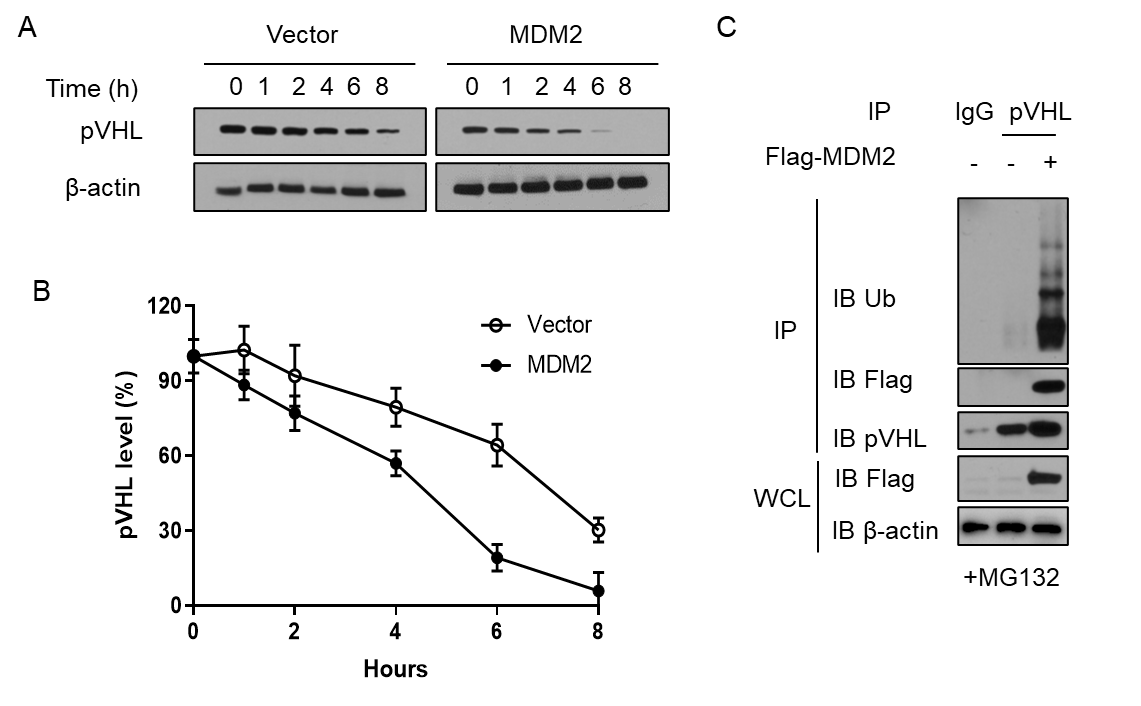


**Figure S2.** MDM2 directly interacts with pVHL and promotes the ubiquitination of pVHL. (A) WERI cells with or without MDM2 overexpression was treated with cycloheximide (CHX) for various time periods and then the level of pVHL in cells was evaluated by western blot. One representative result out of 3 is shown. (B) The western blot results were quantitated by Image J and plotted as mean +/- SD of 3 independent experiments. (C) WERI cells were first overexpressed with MDM2, and then cells were lysed and pVHL was immune-precipitated by pVHL antibody. MDM2, pVHL and ubiquitinated pVHL in the complex were examined by western blot. One representative result out of 3 is shown. IP: immunoprecipitation; WCL: whole cell lysate; IB: immunoblotting.
